# An Au Nanofilm-Graphene/D-Type Fiber Surface Plasmon Resonance Sensor for Highly Sensitive Specificity Bioanalysis

**DOI:** 10.3390/s20040991

**Published:** 2020-02-12

**Authors:** Xiangtai Xi, Jihua Xu, Shuanglu Li, Jingyi Song, Wen Yang, Yang Sun, Shouzhen Jiang, Yanshun Han, Xiuwei Fan

**Affiliations:** 1 Collaborative Innovation Center of Light Manipulations and Applications in Universities of Shandong School of Physics and Electronics, Shandong Normal University, Jinan 250014, China; 2017020511@stu.sdnu.edu.cn (X.X.); xujihua23@163.com (J.X.); 19862176156@139.com (S.L.); sjywywysjy@163.com (J.S.); wenyang0@126.com (W.Y.); 2017020496@stu.sdnu.edu.cn (Y.S.); jiang_sz@126.com (S.J.); 2 Shandong Key Laboratory of Medical Physics and Image Processing & Shandong Provincial Engineering and Technical Center of Light Manipulations, Jinan 250014, China; 3 Qilu Institute of Technology, Jinan 250200, China

**Keywords:** surface plasmon resonance biosensor, D-type plastic fiber, Au-graphene structure, high sensitivity, specificity detection

## Abstract

A highly sensitive Au-graphene structure D-type fiber surface plasmon resonance biosensor is presented in this study to specifically detect biomolecules. The method of growing graphene is employed directly on the copper, and then a gold film of optimum thickness is sputtered, and the copper foil is etched to obtain the structure. This method makes the contact closer between the gold layer and the graphene layer to improve surface plasmon resonance performance. The performance of this type of surface plasmon resonance (SPR) sensor has been previously verified both theoretically and experimentally. With the proposed Au-graphene structure D-type fiber biosensor, the SPR behaviors are obtained and discussed. In the detection of ethanol solution, a red shift of 40 nm is found between the refractive index of 1.3330 and 1.3657. By calculation, the sensitivity of the sensor we designed is 1223 nm/RIU. Besides, the proposed sensor can detect the nucleotide bonding between the double-stranded DNA helix structures. Thus, our sensors can distinguish between mismatched DNA sequences.

## 1. Introduction

Recently, surface plasmon resonance (SPR) technology has aroused huge attention because of its application in the detection of various physical, chemical and biological parameters [1,2]. The oscillations of free electrons at the metal-dielectric interface are called surface plasmons, and they can be photoexcited in thin films of nanostructures [3]. When the total reflection of light occurs at the interface between the medium and the metal film, the evanescent wave will cause the free electrons on the metal surface to form the surface plasma [4]. When the evanescent wave matches the surface plasma, resonance occurs and the light is absorbed by the plasma on the metal surface. As an extremely important spectral analysis technique, the SPR sensor has attracted more and more attention. It can effectively detect small changes in the refractive index (RI) of surrounding media and provide a visual representation of changes or formant changes [4,5].

Compared with the conventional prism-based sensors, the fiber-optic SPR sensor is particularly significant because of its integrated device, easy operation and excellent flexibility for remote detection and immune electromagnetic interference [5,6]. As a component of the SPR sensor, the optical fiber has taken up a prominent position because of its advantages, e.g., high sensitivity to RI changes and ease of manufacture, regulation and control. Thus far, great efforts have been made to improve the performance of fiber optic SPR sensors. A large number of different structures of optical fibers have been studied, e.g., partially uncoated [7], side polished, tapered fiber [8], photonic crystal fiber, D-shaped fiber [9,10,11,12] and U-bend fiber [13,14]. Based on some special advantages, D-shaped fibers have great advantages, e.g., it can easily obtain a large evanescent field for effective sensing applications [6]. To make our structure easy to transfer, the D-type fiber structure was applied here. The conductive film in the fiber generated total reflection at the interface between the core and the cladding. Most of the energy was concentrated in the core, and a part of the energy penetrated the cladding and the external environment. The closer the core is to the external environment, the more energy will penetrate, and the interaction between the outside and the fiber will be stronger. Since the D-type fiber structure has non-circular symmetry, the core is closer to the external environment. Thus, the D-type fiber can be applied to many occasions, e.g., energy coupling, RI sensing and bending sensing. In recent years, nanostructures composed of noble metals such as gold and silver, have been proposed as well as their combinations as special sensitive layers [10]. However, because of the poor chemical stability of some metals, their adsorption capacity is weak. The weak chemical modification ability of these sensitive layers often limits their practical application [15].

In recent years, two-dimensional (2D) nanomaterials have been widely studied because of their potential applications in transistors and opto-electrical converters [16,17,18]. Graphene is a single layer sp2 carbon network disposed of in a perfect cellular lattice [13,19]. Since its being discovered in 2004, it has been one of the most extensively studied 2D nanomaterials [16,20]. Furthermore, the sensitivity of the SPR senor on employing only a single metal film is low [11]. When a graphene layer is deposited on a metal film or metal nanoparticles (e.g., Au or Ag), the metal/graphene interface is strongly coupled because of the effective charge transfer [21]. Graphene exhibits a relatively large surface area (nearly 2630 square meters g^−1^), which increases the area of the contact with analyte, allowing it to absorb more molecules [18,19]. Molecular enrichment of graphene stabilizes the output signal [22,23]. Graphene shows excellent photoelectric properties and adjustable biocompatibility [24]. these advantages of graphene can be used to make more sensitive SPR sensors. Tae-sung Kim’s group reported a D-shaped fiber optic SPR sensor which coated only with graphene as a sensitive and studied the redshift of the biomolecular binding [25]. Subsequently, to investigate the stability of the SPR sensor, Galatus et al. introduced an SPR biosensor, and this sensor is made by transferring graphene onto a multilayer substrate of plastic optical fiber (POF) [26]. The graphene on the metal surface can effectively prevent the oxidation of the metal film, thus ensuring the stability of the sensor [27,28].

Over the past decades, SPR based biosensors have aroused increasing attention because of their important role in real-time and level-free detection [3,29]. Inductive DNA hybridization is a crucial technique in medical diagnosis, which can identify whether the nucleotide bonds are in the right direction [1,18]. In the field of biosensors, SPR technology has taken a place due to its strong sensing ability, compact structure, light weight, good repeatability and reliable DNA hybridization performance [18]. A highly sensitive Au-graphene structure D-type fiber SPR biosensor is developed here to detect DNA hybridization. First, the graphene is developed directly on the bare copper, the gold film of the optimum thickness is sputtered, and the copper foil is etched with FeCl3 solution to obtain the structure of the Au-graphene layer. This method makes the contact closer between the gold layer and the graphene layer. With the proposed Au-graphene structure D-type fiber biosensor, the SPR behaviors are obtained and discussed. In the detection of ethanol solution, a red shift of 40 nm is found between the RI of 1.3330 and 1.3657. By calculation, the sensitivity of the sensor we designed is 1223 nm/RIU. Besides, due to its ability to sense nucleotide binding between double stranded DNA (dsDNA) helical structure, the sensor is able to detect binding between the target DNA (tDNA) and probe DNA anchored to Au-graphene compounds and provide the possibility of distinguishing single base mismatches.

Besides, in order to investigate the practical application of our SPR sensor, we also studied the specificity of the sensor. In optical methods, label-free SPR is a convenient tool for DNA binding detection [19,30]. DNA hybridization refers to a biomolecular method for obtaining genetic differences between organisms by evaluating the genetic relationships between DNA sequence libraries [15]. DNA hybridization has important theoretical value and practical significance in gene screening and detection, disease biomarkers, transcriptional profiling and discovery of single nucleotide variation [15].

## 2. Materials and Methods

### 2.1. Production of D-Type Fiber

**Figure 1:**
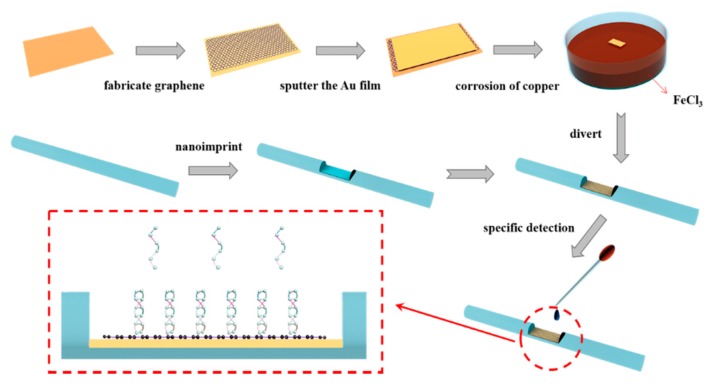
The schematic representation of the preparation procedure of the Au-graphene structure D-type fiber surface plasmon resonance (SPR) biosensor.

Figure 1 schematically illustrates a method of synthesizing the D-type fiber optic sensor. As shown in the figure, we first took a bare POF and wiped off the surface covering with acetone (The length of the fiber was about 15 cm and the diameter was 1 mm). The POF was fixed to a clean glass slide, a 1.5 cm long D-shaped area was roughly cut on the POF with a scalpel, and the depth of the D-type area was close to the radius of the bare fiber. The cutting D-type area was processed with nano-imprint technology to smooth the cutting D-type area. The principle of the nanoimprint device is to make a heating block of the same length as the D-type zone, and the heating block can be accurate to the smoothness of the nanometer scale. We used a fixing device to fix it in the initially cut fiber d-type area and heat it to 120 °C for nanoimprinting. Using a reasonable temperature of 120 °C could not only ensure that the heating temperature can make the D-type zone of the fiber smooth enough, but also did not destroy the morphology of the fiber because of the excessive temperature. We cooled the D-type fiber to ambient temperature and used alcohol and deionized water to remove the residue.

### 2.2. Preparation of Graphene-Gold Layer Structure and Synthesis of Novel D-Type Fiber Sensor

We took a piece of copper foil of 0.5 cm × 1.5 cm size. First, to remove the stain, the foil was soaked three times with acetone for 15 min each time. Subsequently, the copper foil was cleaned three times with alcohol for 15 min each time. Finally, the foil was ultrasonically cleaned three times with deionized water for 10 min each time. The single layer of graphene was grown on clean copper by a chemical vapor deposition system, and the obtained optimum thickness of the Au film was calculated by sputtering on the graphene using a magnetron sputtering system. The copper foil with graphene and Au film was suspended in a suitably configured FeCl_3_ solution to protect the integrity of the graphene and gold layers, and this foil was allowed to stand for 4 h. After waiting for the copper foil to completely corrode, the resulting Au-graphene layer structure was transferred to the D-type region (60 °C for 30 min) of the fabricated D-type fiber using a transfer method to obtain the sensor we wanted to manufacture. The entire sensor production flow chart is shown in Figure 1.

### 2.3. Optical Characterization and Measuring Instruments

**Figure 2:**
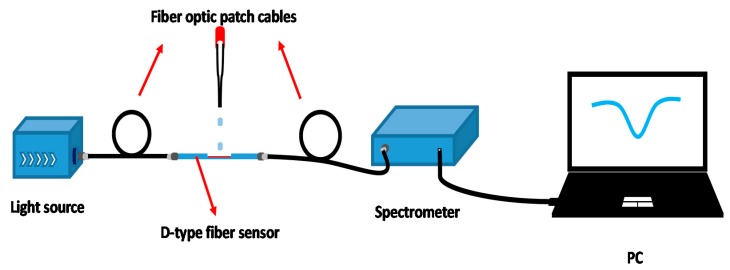
The diagram for the experimental device used in the D-type fiber optic biosensor with Au-graphene structure.

As shown in Figure 2, our experimental device was divided into four sections, successively for the light source, D-type fiber optic sensor, spectrometer (model PG2000) and PC. The light source was used to provide a beam of stable intensity with a wavelength of 380–780 nm. The sensor used the optical fiber patch cord to connect the two ends of the light source and spectrometer respectively. When the D-type fiber sensor was immersed in ethanol solution with different concentrations, the change of RI in the external environment led to the change of light intensity in the fiber. We collected the output signal and recorded the transmission spectrum. At last, Morpho software installed on the PC was used to process the data recorded.

Scanning electron microscopy (SEM) was used to characterize the surface morphology of the D-zone of the sensor. The Raman spectrometer was used to collect the Raman spectra in the D-zone of the sensor to determine the presence of graphene and biomolecules. The laser wavelength used was 532 nm, the integration time was 4 s, and the effective power of the laser source was maintained at 50 mW.

### 2.4. The Specific Detection of Biosensor

To detect the specificity of the sensor, the previously prepared sensor was used as a sample, and the probe DNA was fixed to the D-type region of the sensor by 1-butyric acid succinimide ester (PBASE). Through the real-time detection of biosensors, we obtained the hybridization kinetics of tDNA and probe DNA. Probe DNA and tDNA were purchased from Sangon Biotech Inc. (Shanghai, China). PBASE, dimethyl sulfoxide (DMSO), and phosphate buffered saline (PBS) were obtained from Sigma-Aldrich (Shanghai, China). The sequence of the probe DNA was 5′-AGT ACA TCA CAA CTA-3′, while the sequence of the complementary tDNA was 5′-TAG TTG TGA TGT ACT-3′, and the mis DNA was 5′-CCT CCA CAG CTC GAG-3′.

Graphene’s properties give it a significant advantage in fixing DNA molecules [10]. The strong π-stack between the graphene and the bases maximizes the affinity of the DNA molecules, allowing them to stick to the graphene surface more firmly [10,19]. Accordingly, the entire molecules can fully interact with the surface plasmon and lead to an increase in local RI, thereby making the normalized transmission spectrum red-shifted.

The following is the specificity detection of the D-type fiber biosensor for DNA hybridization. We used PBASE as an adhesive to attach the probe DNA to the surface of graphene so that we could fix the probe DNA without creating defects [15]. First, we dissolved the PBASE powder in DMSO to make the binder we need. 100 mM ethanolamine was used to deactivate the excess reactive groups of PBASE to prevent possible nonspecific binding events, then it was rinsed by DI water [31,32,33]. The prepared PBASE was uniformly applied to the sensor D-type region at ambient temperature, and it was allowed to stand for 4 h while waiting for PBASE to bond with the graphene of the D-type region. The PBASE was then washed in turn with DMSO, ethanol, and deionized water to remove the PBASE that had not yet been attached to the graphene. A π-π stack was established between the graphene hexagon ring and the PBASE fluorenyl group so that the PBASE was fixed on the surface of the graphene to form an adhesive [19]. Next, the sensor D-type region with PBASE as a fixed substrate was uniformly suspended at ambient temperature with a solution containing 2 mM of the probe DNA. The D-zone of the sensor was allowed to static for 4 h, which could ensure sufficient reaction time between the probe DNA and PBASE so that the probe DNA could be firmly fixed on the PBASE. The reason is that the butanediimide group of PBASE extended from the surface of graphene and connect to the amine group of the probe DNA, so that the probe DNA could be fixed. The PBS solution was used sequentially, and deionized water removed unreacted probe DNA. Finally, different concentrations of full-complementary DNA (FC DNA) were used to observe the normalized transmission spectrum of the fiber sensor. The non-complementary DNA (NC DNA) was also selected to compare the normalized transmission spectra with the complementary FC DNA.

## 3. Results and Discussion

**Figure 3:**
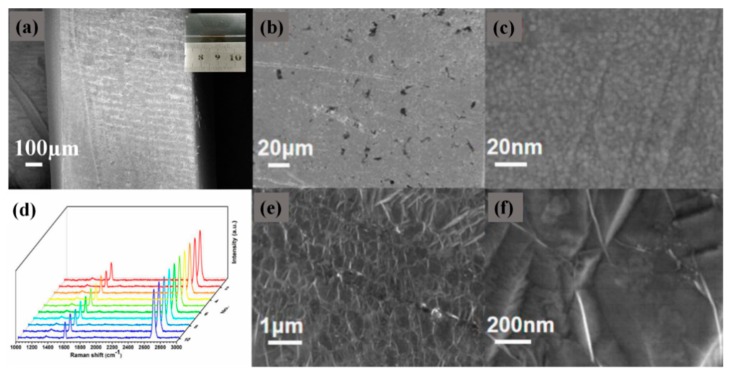
(**a**) The SEM of new D-shape area morphology. (**b**) The SEM image of the D-type area. (**c**) The SEM image of the gold film. (**d**) 10 groups of fixed-point scanning Raman spectra on the same sample. (**e**) The SEM image of grown graphene. (**f**) The SEM image of graphene folds after magnification.

Figure 3 shows the SEM image of the D-type region of the fabricated Au-graphene structure bio-fiber sensor. Figure 3a is the SEM diagram of the newly made D-type fiber D-type region, and the small diagram in the upper right corner is the physical diagram of our sensor. From the figure we can see that the D-shaped area was relatively flat, which was more conducive to our experiment. Figure 3b is a front SEM image of the D-shaped zone. It is suggested from the figure that our structure was directly transferred by the fishing method, which caused partial tearing of the Au-graphene layer, but the overall structure was relatively good. Figure 3c is an enlarged image, and it is suggested from the figure that the gold film was relatively tight overall. To ensure that magnetron sputtering does not cause mass damage to graphene, we performed a large-area fixed-point scan of the sample, selecting ten points in the same composite structure sample. Figure 3d is the Raman spectrum of these ten sets of data. From the figure we can see that there is only a small amplitude between the ten sets of data, and the Raman peak in the figure shows that our graphene still had a high quality. Thus we can prove that the gold film produced by using the magnetron sputtering device will not cause great damage to the graphene layer. Figure 3e is an SEM image of the produced Au-graphene structure, and it is suggested from the figure that the grown graphene had a good alignment structure. Figure 3f is an enlarged image, showing that there are more obvious wrinkles, which proves the existence of graphene. From the SEM image we obtained, we can see that the D-shaped structure we prepared was relatively intact, and the advantage is that it directly sputtered a layer of gold on the graphene layer to ensure better contact between the graphene layer and the gold film. This is more conducive to us to realize the SPR.

**Figure 4:**
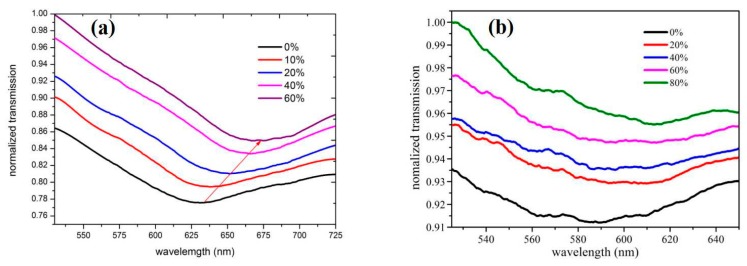
(**a**) Normalized transmission spectrum of Au-graphene structure D-type fiber sensor in alcohol solutions at different concentrations. (**b**) The normalized transmission spectrum of D-type fiber sensor with only a gold film structure in alcohol solution at different concentrations.

To assess the performance of the sensors developed here and to verify that SPR is better represented, the normalized transmission spectra of the sensors were collected in an ethanol solution with an RI of 1.3330 to 1.3657, as shown in Figure 4a. It is suggested that the transmission peak intensity gradually increased with the increase in RI. Besides, the position of the SPR band is red-shifted, and the red shift wavelength is nearly 40 nm. A D-type fiber sensor plated with an optimal thickness of gold was also developed only by magnetron sputtering. Figure 4b shows the normalization transmission spectrum of this sensor in alcohol solution with the RI of 1.3330 to 1.3657. By comparing the differences between Figure 4a,b, a deeper SPR impregnation after the addition of graphene was achieved, and the red shift of the band position after the addition of graphene was larger than that without graphene. Furthermore, the light rate showed a significant drop. Obviously, the sensitivity and linearity between the transmittance and RI in Figure 4a were higher than those in Figure 4b. It can be attributed to the relatively high surface-to-volume ratio of graphene, which allowed it to absorb more molecules with the analyte. With the increase in the concentration of the ethanol solution, the tunable RI of the letters led to a more pronounced change in absorbance. Graphene has good molecular enrichment, which leads to better monitoring of RI changes in the graphene sensing region. The sensitivity of our newly prepared sensor was higher because of the tight coupling at the Au-graphene interface and the high efficiency of charge transfer, which led to greater enhancement of the electric field at the nanometer interface. Besides, graphene has favorable biocompatibility and can assimilate more molecules. Moreover, the structure prepared here was direct magnetron sputtering gold on graphene, improving the graphene and gold layers in the structure here. With sufficient fit, the SPR sensor had good sensitivity.To confirm the sensing performance of the designed SPR sensors, we compared the detected sensitivity with other types of fiber SPR sensors reported in the literature in Table 1 [10,13,34]. By comparing the data, we can conclude that our proposed sensor had higher sensitivity. 

**Figure 5:**
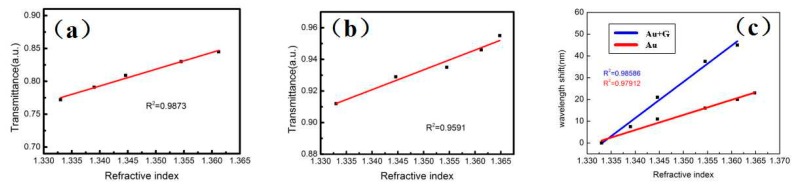
(**a**) Linear relationship between the lowest transmittance of the normalized transmission spectrum of the Au-graphene structure of the D-type fiber sensor under different concentrations of alcohol solution and the RI of the corresponding alcohol solution. (**b**) The linear relationship between the lowest transmittance of the normalized transmission spectrum of the D-type fiber sensor of the gold film structure and the RI of the corresponding alcohol solution in different concentrations of alcohol solution. (**c**) The linear relationship between the red shift of the normalized transmission spectrum and the RI of the corresponding alcohol solution for two D-type fiber sensors under different concentrations of alcohol solution.

Figure 5 is a linear plot of normalized transmission spectra plotted against two D-type fiber optic sensors. Figure 5a shows a linear relationship between the lowest transmittance of the normalized transmission spectrum of the D-type fiber sensor of the Au-graphene structure under different concentrations of alcohol solution and the RI of the corresponding alcohol solution. It is suggested from this figure that the linear relationship is 0.9873, and the linear relationship is good. Figure 5b shows a linear relationship diagram of a D-type fiber sensor with only a gold film structure, and its linear relationship was 0.9591, slightly inferior to that of graphene. Figure 5c is a linear relationship between the wavelength red shift of the normalized transmission spectrum of the two D-type fiber sensors under different concentrations of alcohol solution and the RI of the corresponding alcohol solution. It is suggested this figure that the linear relationship between the wavelength red shift and the RI of the D-type fiber sensor incorporating graphene is 0.98588, higher than 0.97912 without graphene. It is concluded that after the addition of graphene, our D-type fiber sensor exhibited a larger wavelength red shift, verifying that it has higher sensitivity and a better linear relationship. In addition, the SPR effect is better due to the direct sputtering of the gold film on the graphene.

**Figure 6:**
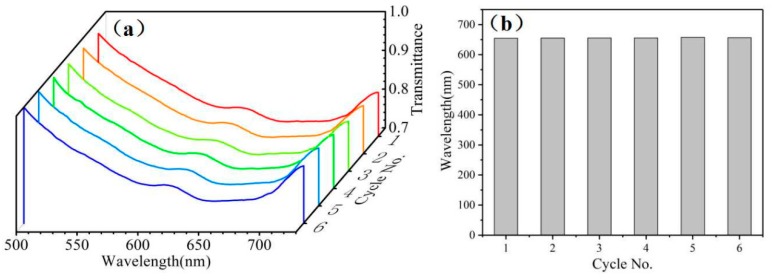
(**a**) D-type fiber sensor normalizes six cycles of transmission spectrum in 1.355 RI alcohol solution. (**b**) Histogram of the wavelength corresponding to the lowest RI of the six normalized transmission spectra.

In addition to sensitivity, stability and repeatability are vital parameters for estimating sensing performance. Under the same conditions, the D-type fiber sensor developed here is studied in the normalized transmission spectrum of the 1.355 RI alcohol solution for six cycles, and the reproducibility of the sensor is achieved under the same RI. As shown in Figure 6a, the spectrum remained basically unchanged. Figure 6b is a histogram of the wavelengths corresponding to the lowest transmittances of the six spectra measured, and it is suggested from the figure that the corresponding wavelengths are substantially unchanged. After six cycles, the good reproducibility of the sensor is proven, suggesting that the D-type fiber sensor developed here exhibits excellent stability and repeatability.

**Figure 7:**
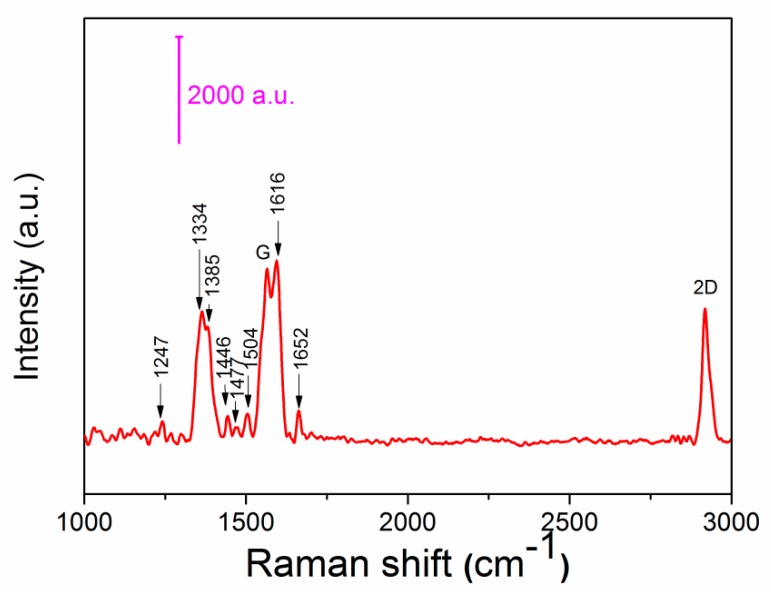
The D-region Raman spectrum of the fabricated biosensor.

Figure 7 shows a Raman diagram of the D-type region of the bio-fiber sensor developed after the above complete process. The characteristic peaks of the added substances were found by referencing the relevant literature [15]. It is suggested from Figure 7, the D-type region of the biofiber sensor finally obtained here had characteristic peaks of all added substances, thereby verifying that all the above substances were contained in our D-type region.

**Figure 8:**
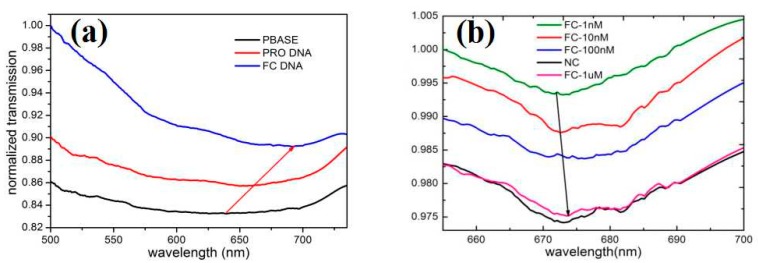
(**a**) The normalized transmission spectra after the addition of PBASE, probe DNA and tDNA to the D-type region. (**b**) The normalized transmission spectra of different concentrations of full complementary (FC) and non-complementary (NC) DNA.

Figure 8a shows the normalized transmission spectra measured after the addition of PBASE, probe DNA and tDNA to the D-type region. It can be seen from the figure that we add the same concentration of PBASE, probe DNA, and tmDNA will produce the red-shift effect compared with the previous one. After the addition of PBASE, due to the doping effect of PBASE, the charge transfer between the pyrene group and graphene was caused, and the charge neutral point voltage moved forward [35]. With the addition of probe DNA, the neutral voltage continued to move forward due to the negatively charged DNA gating effect. Thus, the normalized transmission spectrum generated a red shift effect [36]. When the probe DNA on the surface was complementary to the two single strands of complementary DNA and formed a dsDNA helix, a complementary hybridization event was defined. There was a slight change in the SPR angle, and hydrogen bonding occurred between the probe DNA and the tDNA strand [19]. Accordingly, the charge in the target molecule was changed significantly, resulting in a red shift phenomenon. Figure 8b shows the effect of different concentrations of FC DNA on the normalized transmission spectra of the sensor and gives the normalized transmission spectra of NC DNA. It is suggested from the figure that with the increase in the concentration of FC DNA, the normalized transmission spectrum also shows a certain red shift phenomenon. It is again verified that hydrogen bonding occurred between the probe DNA and the FC DNA strand, and the charge in the molecule was changed significantly, and the higher the FC DNA concentration, the charge would be changed more significantly, resulting in a red shift. The normalized transmission spectrum of NC DNA exhibited little red shift, suggesting that the base mismatch event of the probe DNA and NC DNA resulted in no significant change in the SPR angle when the probe molecule is used. By entering the mismatched target, hydrogen bonding did not occur between the probe DNA and the tDNA strand due to mismatch [19]. Thus, there was no change in the target molecule.

**Figure 9:**
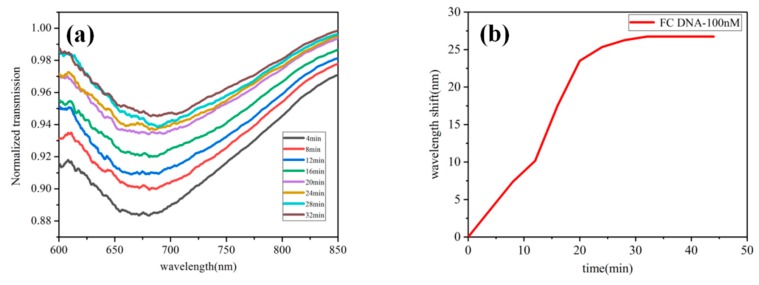
(**a**) The normalized projection spectra for real-time monitoring of FC DNA at 100 nM concentrations. (**b**) The function of wavelength shift over time.

To further evaluate the performance of the sensor in DNA detection, we used this sensor for real-time detection of FC DNA with 100 nm concentration and recorded the normalized projection spectra. We can see in Figure 9a that the transmission spectrum gradually changed as DNA hybridization proceeded. Figure 9b makes the function of wavelength shift with time, showing the behavior of nonlinear change. The normalized projection spectrum produced a red shift due to the binding of FC DNA to probe DNA. The red shift increased rapidly from 0 to 20 min, tended to be gentle at 20 to 25 min, and the wavelength shift tended to remain constant at more than 25 min. This means that the molecular reaction kinetics reached saturation.

## 4. Conclusions

To sum up, a highly sensitive Au-graphene structure D-type fiber SPR biosensor is presented here. The Au-graphene structure obtained here makes the contact more closely between the gold layer and the graphene layer. Graphene makes the designed sensor higher in sensitivity. The proposed sensor exhibits excellent stability and repeatability. The designed sensor can detect nucleotide bonding between the dsDNA helix structures, and therefore mismatched DNA sequences can be detected.

## Figures and Tables

**Table 1:** Comparison of sensitivity with other fiber optic sensors.

Sensor Type	Material	Sensitivity (nm/RIU)	Reference
D-shape fiber	Au/graphene	413.79	[10]
D-shape fiber	Au nanostars	580	[34]
U-bent POF	AgNPs/graphene	700.3	[13]
D-shape POF	Au/graphene	1223	Our work
